# Testing a model of association between patient identified problems and responses to global measures of health in low back pain patients: a prospective study

**DOI:** 10.1186/1477-7525-7-74

**Published:** 2009-08-05

**Authors:** Ricky Mullis, Martyn Lewis, Elaine M Hay

**Affiliations:** 1 Arthritis Research Campaign National Primary Care Centre, Keele University, Keele, UK

## Abstract

**Background:**

Self-rated health status has been shown to be related to physical function. Therefore, changes in functional ability should be associated with changes in general health. However, functional needs may vary greatly between individuals. The purpose of this study was to propose and test a model of association between patient identified functional problems and responses to global measures of health in low back pain patients.

**Methods:**

Participants in a low back pain clinical trial were followed up for 12 months. A series of analyses were undertaken using the Jonckheere-Terpstra test and chi-square for trends to determine the associations between two individualised items related to function, and measures of "overall improvement in condition", "general health status" and performance of "usual activities".

**Results:**

Significant associations between responses to the five items were found. Performance of usual activities is significantly associated with ratings of general health status (p < 0.001) and overall condition of the back (p < 0.001). The extent to which the patient identified problems influence an individual's perception on multi-task performance is dependent upon the degree of difficulty and level of importance attached to these problems.

**Conclusion:**

The relationship between patient identified problems and responses to global measures of health is complex. The explanatory model proposed here may improve our understanding of these interactions.

**Trial Registration:**

ISRCTN 32765488

## Background

### Theory underpinning possible associations between responses

Measuring the success of an intervention to change a patient's health is central to both research and clinical practice. Self-rated health status has been shown to be related to a number of distinct constructs including physical function, medication use and mental well-being [1-3]. Wilson and Cleary [4] described a five level classification scheme for different measures of health outcome, ranging from biological and physical factors, symptoms, functioning, general health perceptions, through to overall quality of life (QoL). Further, they proposed a causal link between the levels, with each becoming "increasingly integrated and increasingly difficult to define and measure". Smith et al. [5] explored a structural model of the determinants of health status and QoL, suggesting that self-evaluation of these two constructs are "determined jointly by assessments of several domains". They hypothesised a cognitive process that involved:

1) identifying the relevant domains comprising the construct

2) determining where one stands on each domain

3) integrating the separate domain judgements into an overall assessment

Self-rated recovery from back pain has been shown to depend upon an individual's cognitive appraisal of the impact of symptoms on their ability to perform meaningful daily activities [6] and functional every day tasks were found to be important outcome markers for patients with musculoskeletal pain [7]. Perceptions about QoL and general health status are also unique to the individual, and determination of the relevant domains that comprise these constructs must take account of what is important at an individual level. Therefore, if function plays a role in how general health is perceived (as suggested by Wilson and Cleary [4]), there should be a clear association between changes in functional ability and changes in general health. Similarly, if specific functional activities are considered an important element of day to day *usual activities*, an association between these items would also be expected. If a link can be proven, then targeting these patient-specific functional tasks as an aim of treatment may lead to improved outcomes.

Individualised or "patient-specific" measures allow the respondent to select the important issues or concerns which affect them the most. They are free from the type of pre-determined items which form the majority of standardised questionnaires, and which may contain elements of little importance to some, whilst omitting items of relevance to others. One criticism of this type of measure is that without standardisation of the items, the scales are not the same in each patient and the numeric scores do not hold a common meaning [8]. However, similar arguments may be levelled when comparisons are made across different fixed item scales which produce very different impressions of health in the same sample [9].

It has been shown that people who suffer with low back pain can readily identify important aspects of their lives that are affected [10]. These ranged from purely functional daily activities (e.g. washing, dressing, moving from one position to another) to areas that affect an individuals' wider role in society and quality of life (e.g. employment and recreational activities). The breadth of problems experienced by people with back pain provides the opportunity to explore the concepts proposed by Wilson and Cleary [4] and Smith et al. [5], and may be illustrated by reference to five items taken from a questionnaire used in a clinical trial [11]. Items one (Q1) and two (Q2) were individualised, requiring the respondent to identify a single difficult or usually enjoyed activity affected by their back pain. Item three (Q3) referred to *usual activities*, which could (amongst others) include those things identified in Q1 and Q2. The theories of Wilson and Cleary [4] and the model described by Smith et al. [5] would suggest that the single activities identified from Q1 and Q2 may act as salient points of reference for individuals when they consider the *usual activities *item, and may influence how they perceive their overall performance of multiple tasks. Similarly, when appraising the broader constructs of general health (Q4) and overall change in condition (Q5) in the form of single item global measures of health status, one of the anchors to which an individual may consciously (or otherwise) refer is their performance of *usual activities*, which could influence their perception of these more complex aspects of health.

**Figure 1:**
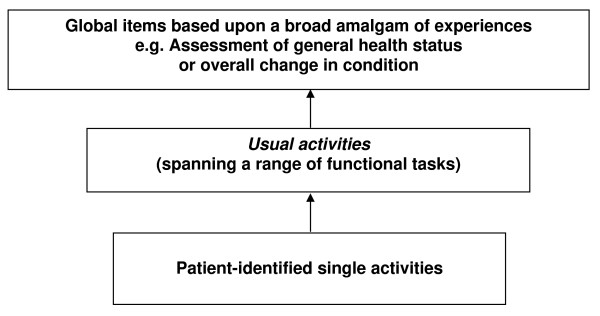
**The structure of a theoretical hierarchy between single activity items and broader constructs of health**.

This proposed link between the five items can be visually depicted as forming a three tier hierarchy. The level at which each item appears within the hierarchy is determined by the breadth of factors taken into consideration in forming a response. Thus, the individualised patient-identified single activity items (Q1 and Q2) form the bottom tier of the structure. The middle tier contains the *usual activities *component of the EuroQol instrument (Q3) [12], as this calls for a response based across a range of functional tasks. Ratings of general health status (Q4) and overall change in condition (Q5) incorporate the broadest constructs of measurement and occupy the top tier. Figure 1 depicts this three tier structure.

**Figure 2:**
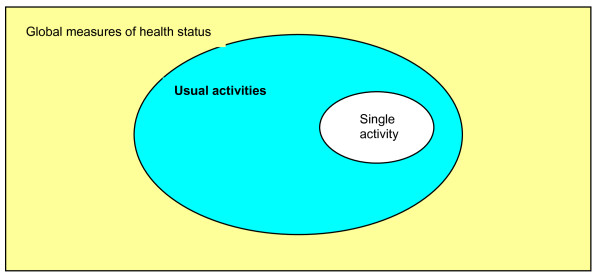
**Venn diagram depicting a simple "sub-group" relationship between the different tiers of the item hierarchy**.

Each layer represents a broader span of considerations and an increasing level of anchor point complexity over that below it. However, it is hypothesised that these tiers are not independent of each other. One possible model of association is that the single activity items in the lowest tier exist as a simple subgroup within the items above. This relationship is depicted in figure 2.

If this were so, then the single activities which individuals identified as important may act as salient points of reference when considering responses to the multi-task *usual activities *item and to broader general health questions. However, the extent to which these single activity items influence the more generic measures may not be quite so simple. Figure 2 suggests that the single activity items form a set area within the space occupied by *usual activities*, which in turn takes up a given area of the larger total space representing the global measures of health status. However, the relative proportion of space that the single activities occupy within the two larger areas differs; and it could also be surmised that the influence that these items have on each of the larger areas will also differ. In this context, this would mean that the single activities (occupying a relatively large proportion of the *usual activities *space) would have a greater impact upon an individual's perception of their ability to perform *usual activities*, than they would on broader general health issues (where the "single activity" occupies proportionately less space).

**Figure 3:**
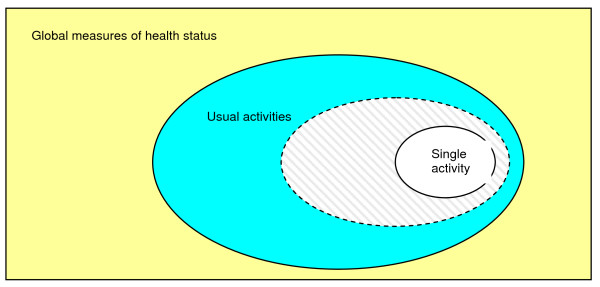
**Venn diagram depicting the variable potential of a single chosen activity to influence responses to items comprising broader constructs**.

Other factors may also influence these relationships. For example, the degree of difficulty associated with these single activities or the importance attached to them may contribute to the strength of these associations. We hypothesised that single chosen activities which are rated higher in difficulty or importance by patients will bear a greater influence on responses to the items composing broader contructs when compared with those rated less difficult or perceived to be of little importance. The variable potential of the single chosen activity to influence responses to more complex items is depicted in figure 3; with more difficult or important single activities (represented by the grey shaded area) occupying a larger proportion of space within the diagram.

The aim of this study was to test this model by undertaking a series of analyses to determine the associations between the two individualised items and measures of "overall improvement", "general health status" and performance of "usual activities". Specifically, the following hypotheses were tested:

**Hypothesis 1 **– *The perceived ability to perform usual activities (Q3) will be positively associated with*

i) the difficulty associated with the single activity (Q1)

and ii) the importance attached to the single activity (Q2)

**Hypothesis 2 **– *The responses given to the broadest construct items (Q4 and Q5) will be positively associated with the responses given to the single activity items (Q1 and Q2)*.

**Hypothesis 3 **– *The responses given to the broadest construct items (Q4 and Q5) will be positively associated with perceived ability to perform usual activities (Q3)*.

**Hypothesis 4 **– *The strength of the association between measures which occupy adjacent tiers of the hierarchical model (described in figure *1*) will be greater than that between the bottom and top tiers*

*i.e. i) Q1 and Q2 will be more strongly associated with Q3 than with Q4 and Q5*;

*and ii) Q3 will be more strongly associated with Q4 and Q5 than will Q1 and Q2*.

## Methods

The hypotheses were tested by analysing patients' responses to five items (Q1 to Q5) used in a self-completed questionnaire in a low back pain trial conducted by Hay et al. [11]. This randomised controlled trial compared the clinical effectiveness, in primary care, of a brief pain management programme delivered by physiotherapists with that of a programme of spinal manual physiotherapy in the treatment of non-specific low back pain of less than 12 weeks duration. The primary outcome was change in self-reported back pain related disability at 12 months. Favourable ethical opinion for this study was gained from North Staffordshire Local Research Ethics Committee (Project No.1123).

**Q1 **During the baseline assessment, the following question was asked:

*Because of your back pain, what one thing do you find the most difficult to do*?

Using a 10 cm visual analogue scale (VAS) participants were then asked to indicate how difficult this thing was (0 = "no difficulty", 100 = "worst imaginable difficulty").

At 12 months follow-up, participants were reminded of the activity which they had selected at baseline, and asked to indicate how difficult it currently was.

**Q2 **At baseline, participants were asked:

*Is there one thing that you really enjoy doing usually that you are unable to do at the moment, because of your back pain*? (Yes/No)

Those who responded "yes" were then asked: *What is this thing that you enjoy and can't do at the moment*? and to indicate how important this was to them on a VAS (0 = "not important", 100 = "very important").

At follow-up, those participants who had identified a *usually enjoyed activity *at baseline were reminded of what they had selected, and asked whether they were now able to do it.

Responses to Q1 and Q2 were unprompted, and recorded as free text. The content of these responses have been reported elsewhere [10].

**Q3 **This item from the EQ-5D [12] relates to problems with "usual activities", with response options "no problems", "some problems" and "unable to perform" *usual activities*.

**Q4 **Participants rated their general health status at baseline and follow-up as either "excellent", "very good", "good", "fair" or "poor".

**Q5 **At follow-up, participants were asked: *In general, how has your back been feeling since you entered this study*?

Response options were "Completely better", "much better", "better", "same", "worse" and "Much Worse".

### Analyses

#### Hypothesis 1

Patients were categorised according to their responses to Q3. The Jonckheere-Terpstra test for ordered alternatives was used to identify trends in patient identified single task difficulty (Q1) and importance scores (Q2) across responses to Q3. This method is considered appropriate when the order of the groups is specified *a priori *[13].

On Q2, the Chi-square test for trend was used to compare the responses to Q3 of patients who at follow-up were subsequently able to take part in their selected usually enjoyed single activity with those who were still not able to do so.

#### Hypothesis 2

Patients were grouped according to their general health status (Q4) and to change in condition (Q5). The Jonckheere-Terpstra test was used to identify trends in patient identified task difficulty (Q1) across responses to Q4 and Q5.

For Q2, chi-square for trends was used to:

i) compare responses to Q4 in patients who were able to identify a specific usually enjoyed activity with those who could not;

ii) compare patients who at follow-up, were able to take part in their selected usually enjoyed single activity with those who were still not able to do so.

#### Hypothesis 3

The Jonckheere-Terpstra test was used to identify trends in general health status (Q4) across reported ability to complete *usual activities*, and trends in "change in condition" (Q5) with changes in ability to complete *usual activities*.

#### Hypothesis 4

Findings from the analyses for hypotheses 1, 2 and 3 will be collated.

## Results and discussion

### Hypothesis 1

The perceived ability to perform usual activities (Q3) will be positively associated with

i) the difficulty associated with the single activity (Q1)

and ii) the importance attached to the single activity (Q2)

*Q1 and Q3 *– Data were available for both items on 396 patients at baseline, and 300 at 12 months follow-up. A summary of these data can be seen in table 1.

**Table 1:** Task difficulty VAS scores categorised by *usual activities *responses at baseline and 12 months

	Task difficulty (VAS)					
	Baseline			12 Months		

*Usual Activities*	n	Median	IQR	n	Median	IQR

No problems	185	61	28	191	1	10

Some problems	181	63	28	100	25	41

Unable	30	74	24	9	71	45

**Figure 4:**
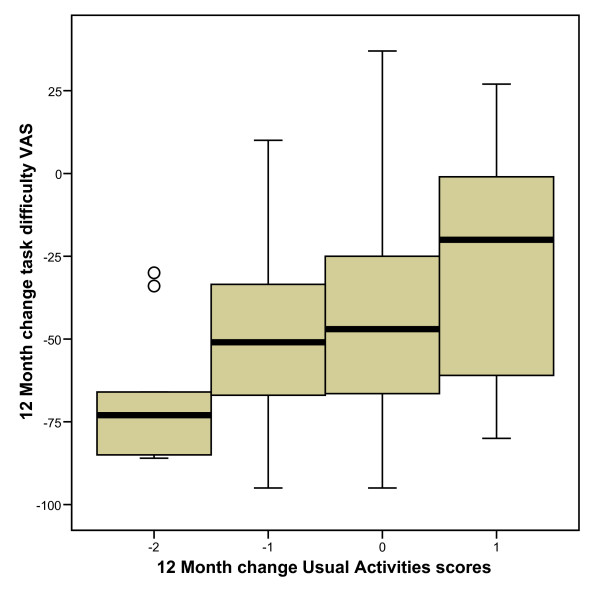
**Boxplot of task difficulty change scores across change in *usual activities *at 12 months**.

Change in task difficulty between baseline and follow-up was categorised according to change in *usual activities *responses and can be seen in figure 4.

The Jonckheere-Terpstra test revealed a significant trend in the order of the median scores of task difficulty across the three *usual activities *groups at baseline (p = 0.015) and at 12 months (p < 0.001), and across change scores (p < 0.001).

*Q2 and Q3 *– Data were available for Q2 and Q3 on 405 patients at baseline, and 311 at follow-up.

At baseline 311 patients (76.8%) were able to specify a usually enjoyed activity that they were prevented from doing. A cross-tabulation of data from Q2 and Q3 is shown in table 2.

**Table 2:** Cross-tabulation showing frequency of responses (%) to the *usual activities *item with identification and importance of a specific single "usually enjoyed activity" and with ability to take part in the single "usually enjoyed activity" at 12 months

Baseline *Usual Activities*	Specific "usually enjoyed activity" identified			
		
	No	Yes		
		
	n (%)	n (%)	Importance (IQR)	Total
No problems	46 (48.9)	142 (45.7)	76 (30)	188

Some problems	45 (47.9)	142 (45.7)	88 (30)	187

Unable	3 (3.2)	27 (8.7)	93 (21)	30

Total	94	311		405

				

12 months *Usual Activities*	12 months "able to take part in usually enjoyed activity"			
		
	no	yes		Total

No problems	21 (42.0)	120 (63.5)		141

Some problems	24 (48.0)	66 (34.9)		90

Unable	5 (10.0)	3 (1.6)		8

Total	50	189		239

The Jonckheere-Terpstra test revealed a significant trend in the order of the median scores of importance of chosen single activity across the three *usual activities *groups (p < 0.001). Chi-square for trends revealed that patients who were subsequently able to take part in their chosen "usually enjoyed activity" at 12 months had less problems doing *usual activities *compared with those who were still unable to take part in the single activity (χ^2 ^= 11.22, p = 0.001).

#### Discussion

The data demonstrates a clear and significant association between performance on the individualised single items and perceived ability to undertake *usual activity*, although the relationships are not simple.

Patients who stated that they were "unable" to perform *usual activities *rated their self-identified "most difficult thing" as harder to do, compared with those patients who had either "no problems" or "some problems" with *usual activities*. Furthermore, there were significant trends in the order of scores of task difficulty across the *usual activities *response categories at baseline and follow-up. Those patients who had improved the most in terms of task difficulty, also showed the greatest change in ability to perform *usual activities*.

A similar pattern emerged in the responses to Q2. Patients who indicated that they had "no problems" with *usual activities *rated the importance of their specific chosen activity lower than those patients who had "some problems" or were "unable to perform" *usual activities*. At follow-up, patients who were subsequently able to take part in their chosen "enjoyed activity" were more likely to have no problems doing *usual activities*, compared with those who were still unable to take part in the single activity.

The evidence presented supports hypothesis one.

### Hypothesis 2

*The responses given to the broadest construct items (Q4 and Q5) will be positively associated with the responses given to the single activity items (Q1 and Q2)*.

*Q1 and Q4 *– Data were available for both measures on 397 patients at baseline, and 300 at 12 months. A cross-tabulation of these data can be seen in table 3.

**Table 3:** Task difficulty VAS scores categorised by general health status at baseline and 12 months

	Task difficulty (VAS)					
	Baseline			12 Months		

General health	n	Median	IQR	n	Median	IQR

Excellent	32	62	26	19	0	2

Very good	137	63	31	110	2	18

Good	171	62	28	112	9	29

Fair	47	68	28	46	35	54

Poor	10	75	23	13	18	61

The Jonckheere-Terpstra test found no significant trend in the order of the median scores of task difficulty across the five general health categories at baseline (p = 0.668) but revealed a significant trend at 12 months (p < 0.001). When the change in task difficulty scores over 12 months were categorised according to change in general health status, the Jonckheere-Terpstra test found no significant trend in the order of the median scores (p = 0.064).

*Q1 and Q5 *– Data were available for both measures on 299 patients at 12 months follow-up.

**Figure 5:**
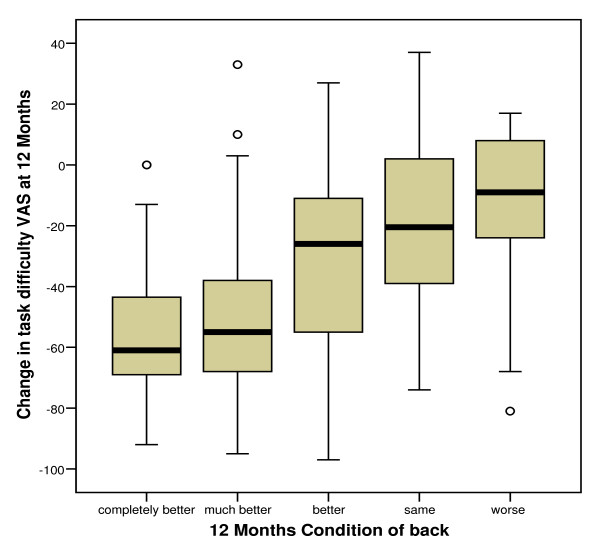
**Boxplot of the distribution of task difficulty change scores according to self reported condition of back at 12 months (scores of "Worse" and "Much worse" are pooled)**.

Change in task difficulty was categorised according to overall condition of back, and can be seen in figure 5.

The Jonckheere-Terpstra test revealed a significant trend in the order of the median scores of change in task difficulty across the "change in condition of back" categories at 12 months (p < 0.001) Patients reporting themselves as completely better recorded the most beneficial change in task difficulty.

Q2 and Q4 – Data were available for both measures on 406 patients at baseline, of which 312 (76.8%) were able to identify a specific "usually enjoyed activity" that they were prevented from doing. At 12 months, data were available on 238 (76.3%) of these original 312 patients. A cross-tabulation of these data can be seen in table 4.

**Table 4:** Cross-tabulation of response frequency (%) on general health status with identification of a specific "usually enjoyed activity" at baseline, and with ability to perform this activity at 12 months

Baseline General health status	Specific "usually enjoyed activity" identified		
		
	no	yes	Total
Excellent	5 (5.3)	27 (8.7)	32

Very good	34 (36.2)	105 (33.7)	139

Good	48 (51.1)	127 (40.1)	175

Fair	7 (7.4)	43 (13.8)	50

Poor	0	10 (3.2)	10

Total	94	312	406

			

12 Months General health status	Now able to perform "usually enjoyed activity"?		Total
		
	no	yes	

Excellent	1 (2.0)	12 (6.4)	13

Very good	10 (20.0)	73 (38.8)	83

Good	19 (38.0)	71 (37.8)	90

Fair	15 (30.0)	25 (13.3)	40

Poor	5 (10.0)	7 (3.7)	12

Total	50	188	238

At baseline, no statistically significant difference was found on general health status between those who could identify a "usually enjoyed activity" and those who could not (χ^2 ^= 0.68, p = 0.406). At 12 months, those who were able to perform their chosen activity rated their general health significantly better than those who were still not able to (χ^2 ^= 14.11, p < 0.001).

*Q2 and Q5 *– Data were available for both measures on 235 patients at 12 months, and a cross-tabulation is shown in table 5.

**Table 5:** Cross-tabulation showing frequency of responses (%) to change in condition of back at 12 months with ability to perform a self-selected "usually enjoyed activity"

12 Months Change in condition of back	Now able to perform "usually enjoyed activity"?		Total
		
	No	yes	
Completely better	6 (12.0)	38 (20.5)	44

Much better	18 (36.0)	101 (54.6)	119

Better	9 (18.0)	22 (11.9)	31

Same	7 (14.0)	18 (9.7)	25

Worse	8 (16.0)	5 (2.7)	13

Much worse	2 (4.0)	1 (0.5)	3

Total	50	185	235

Chi-square for trends showed that those who were subsequently able to participate in their chosen single activity rated their change in condition significantly better than those who were still not able to (χ^2 ^= 17.75, p < 0.001).

#### Discussion

The association between responses to the single activity items and perception of general health is equivocal. At baseline, general health was not associated with the degree of difficulty experienced with self-selected tasks. Conversely, at 12 months a significant trend was evident. Similarly, at baseline there was no difference in the distribution across health status categories between those who could and those who could not identify a specific "usually enjoyed activity". However, at follow-up, patients who were subsequently able to take part in their chosen activity rated themselves to be in better general health than those who were still unable to participate. The inconsistency of the evidence relating responses on the two individualised items to general health status suggests that any likely association is weak.

Conversely, an improvement in the overall condition of a patient's back was associated with improvements in performing their chosen single activities. At follow-up, most patients experienced less difficulty completing their cited task, and there was a significant trend associated with improvement in the overall condition of their back. Similarly, those who had returned to this activity within the follow-up period, perceived their condition to have improved significantly more than those who had not.

Therefore, the data presented here do not fully support hypothesis 2. Based on the evidence available, there may be a stronger association between the single activity responses and overall condition of the back than with perception of general health status.

### Hypothesis 3

*The responses given to the broadest construct items (Q4 and Q5) will be positively associated with perceived ability to perform usual activities (Q3)*.

**Figure 6:**
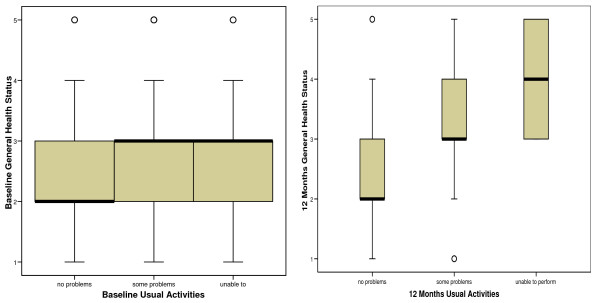
**Boxplots of the distribution of health status scores at baseline and 12 months according to *usual activities *responses**.

*Q3 and Q4 *– Data were available for both measures on 405 patients at baseline and 330 at 12 months. The distribution of health status scores across *usual activities *responses is shown in figure 6.

The Jonckheere-Terpstra test revealed a significant trend in the order of the median scores of general health status across the three *usual activities *groups at baseline (p < 0.001) and at 12 months (p < 0.001), and also with change scores across these two measures (p < 0.001).

**Figure 7:**
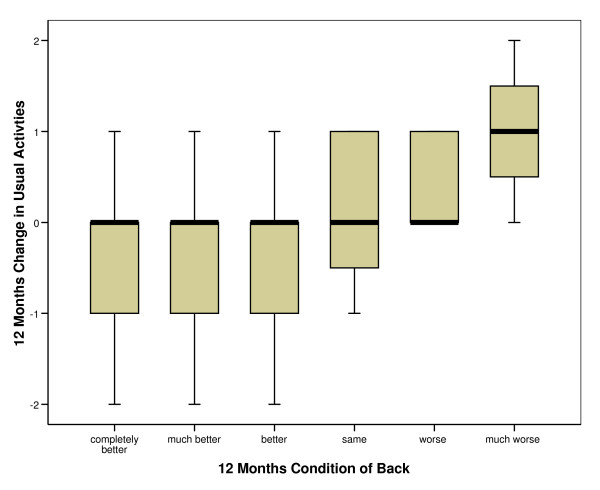
**Boxplot showing change on *usual activities *with change in condition of back at 12 months**.

*Q3 and Q5 *– Data were available for both measures on 311 patients at 12 months. The distribution of usual activities change scores across change in condition of back is shown in figure 7.

The Jonckheere-Terpstra test revealed a significant trend in the order of the median scores of change in condition of back across change in *usual activities *at 12 months (p < 0.001).

#### Discussion

There were significant associations between perceived ability to complete *usual activities*, and both general health status and change in overall condition of back. When grouped according to *usual activities *responses, there were significant differences in general health status both at baseline and follow up, as well as significant positive trends on change scores across both sets of measures.

The data presented here therefore support hypothesis three.

### Hypothesis 4

*The strength of the association between measures which occupy adjacent tiers of the hierarchical model (described in figure *1*) will be greater than that between the bottom and top tiers*

*i.e. i) Q1 and Q2 will be more strongly associated with Q3 than with Q4 and Q5*;

*and ii) Q3 will be more strongly associated with Q4 and Q5 than will Q1 and Q2*.

#### Discussion

The evidence presented above for hypotheses one, two and three support hypothesis four, although not unequivocally. Responses to both of the single activity items were associated with ability to perform *usual activities*. These items sit in adjacent layers of the hierarchical model depicted in figure one. However, the associations between Q1 and Q2 (occupying the bottom tier) and those in the top tier (Q4 and Q5) are less consistent; whereas the ability to perform *usual activities *(middle tier) is more strongly associated with the more complex items occupying the top tier.

Overall, the evidence tends to support hypothesis four.

## Conclusion

The relationship between these layers is complex, and the data more readily fits the model depicted in the Venn diagram of figure three, supporting the theory describing the variable potential of the single activity items to influence responses to the items comprising broader constructs. The degree of difficulty or level of importance ascribed to the single activities was significant in how patients responded on the more global measures of health status. The single items which patients chose may have contributed (along with a range of other functional tasks) to their perception of completing *usual activities*. This in turn fed in (to a lesser extent) to their perception of general health status and the overall condition of their back. These associations are in keeping with the causal link theory [4], and sit within the structural model proposed by Smith et al. [5] whereby relevant domains are identified, appraised and then integrated to provide an overall assessment of health status.

Functional tasks that affected every day living were found to be the most important outcome markers for patients with musculoskeletal pain [7]. Hush et al. described a link between self-rated recovery from back pain and ability to perform meaningful daily tasks [6]. However, both of these reports were based upon qualitative interviews. This study adds to the body of knowledge by demonstrating a statistical association between functional every day tasks and more global measures of health status. The development of fixed-item instruments that measure functional health outcome should take consideration of the importance as well as the difficulty of completing the tasks selected.

Furthermore, Carnes & Underwood concluded that "Treatment progress can be more meaningfully monitored by using patient determined goals, rather than clinical outcomes" [7]. The types of functional activity that patients identified as important in Q1 and Q2 (described in detail by Mullis et al [10]) could easily be included within a clinical assessment and translated into treatment goals. We have shown that achievement of these activities is associated with a perceived improvement in overall condition, and that this association is stronger when these activities are more important to the individual. Work to assess whether targeting specifically these goals can lead to further improvements in outcome is recommended.

## Limitations

These analyses were performed on data collected during a randomised clinical trial of treatments for low back pain [11]. Although the breadth of problems experienced by people with back pain provided the opportunity to explore associations between the different constructs, the sample size was not powered to test the hypotheses, which may present the possibility of a type 2 error. However, most of the analyses led to the dismissal of the null hypothesis, thereby suggesting that the risk of this was relatively small.

The trial participants were "a defined subset of all primary care consulters with non-specific low back pain – those consulting with a current episode duration of less than 12 weeks" [11]. Such patients account for approximately one in five of all primary care consulters with low back pain [14]. It is possible that persistent chronic low back pain sufferers may respond differently to these questions.

## The main findings of this paper are summarised in the following points

• There are significant associations between responses to the five questions.

• The extent to which the single activities identified by patients on the individualised questions influence their perception on multi-task performance is dependent upon the degree of difficulty and level of importance attached to these.

• Perception of the performance of *usual activities *has an effect upon ratings of general health status and overall condition of the back.

• Ability to undertake the single activity items is associated with improvement in overall condition of the back, but not general health status.

• The explanatory model proposed may improve our understanding of these complex relationships.

## Competing interests

The authors declare that they have no competing interests.

## Authors' contributions

RM contributed to the design of the explanatory model and the study, data collection and analysis, and writing of the manuscript. ML contributed to the design of the study, data analysis and writing of the manuscript. EH contributed to the design of the study, interpretation of the findings and writing of the manuscript.

## References

[B1] Benyamini Y, Idler EL, Leventhal H, Leventhal EA (2000). Positive affect and function as influences on self-assessments of health: expanding our view beyond illness and disability. Journals of Gerontology Series B, Psychological Sciences and Social Sciences.

[B2] Krause NM, Jay GM (1994). What do global self-rated health items measure?. Medical Care.

[B3] Mitrushina MN, Satz P (1991). Correlates of self-rated health in the elderly. Aging (Milano).

[B4] Wilson IB, Cleary PD (1995). Linking clinical variables with health related quality of life. A conceptual model of patient outcomes. Journal of the American Medical Association.

[B5] Smith KW, Avis NE, Assmann SF (1999). Distinguishing between quality of life and health status in quality of life research: A meta analysis. Quality of Life Research.

[B6] Hush JM, Refshauge K, Sullivan G, De Souza L, Maher CG, McAuley JH (2009). Recovery: What does this mean to patients with low back pain?. Arthritis & Rheumatism (Arthritis Care & Research).

[B7] Carnes D, Underwood M (2008). The importance of monitoring patient's ability to achieve functional tasks in those with musculoskeletal pain. International Journal of Osteopathic Medicine.

[B8] Jolles BM, Buchbinder R, Beaton DE (2005). A study compared nine patient-specific indices for musculoskeletal disorders. Journal of Clinical Epidemiology.

[B9] Beaton DE, Hogg-Johnson S, Bombardier C (1997). Evaluating changes in health status: reliability and responsiveness of five generic health status measures in workers with musculoskeletal disorders. Journal of Clinical Epidemiology.

[B10] Mullis R, Barber J, Lewis M, Hay M (2007). The ICF *Core Sets *for low back pain: Do they include what matters to patients?. Journal of Rehabilitation Medicine.

[B11] Hay EM, Mullis R, Lewis M, Vohora K, Main CJ, Watson P, Dziedzic KS, Sim J, Minns Lowe C, Croft PR (2005). Comparison of physical treatments versus a brief pain management programme for back pain in primary care: a randomised clinical trial in physiotherapy practice. Lancet.

[B12] EuroQol Group (1990). EuroQol – a new facility for the measurement of health related quality of life. Health Policy.

[B13] Siegel S, Castellan NJ (1988). Nonparametric statistics for the behavioral sciences.

[B14] Dunn KM, Croft PR (2005). Classification of low back pain in primary care: using "bothersomeness" to identify the most severe cases. Spine.

